# Exercise-Induced Neuroprotection in the *Spastic* Han Wistar Rat: The Possible Role of Brain-Derived Neurotrophic Factor

**DOI:** 10.1155/2015/834543

**Published:** 2015-02-01

**Authors:** Brooke H. Van Kummer, Randy W. Cohen

**Affiliations:** Department of Biology, California State University, 18111 Nordhoff Street, Northridge, CA 91330-8303, USA

## Abstract

Moderate aerobic exercise has been shown to enhance motor skills and protect the nervous system from neurodegenerative diseases, like ataxia. Our lab uses the *spastic* Han Wistar rat as a model of ataxia. Mutant rats develop forelimb tremor and hind limb rigidity and have a decreased lifespan. Our lab has shown that exercise reduced Purkinje cell degeneration and delayed motor dysfunction, significantly increasing lifespan. Our study investigated how moderate exercise may mediate neuroprotection by analyzing brain-derived neurotrophic factor (BDNF) and its receptor TrkB. To link BDNF to exercise-induced neuroprotection, mutant and normal rats were infused with the TrkB antagonist K252a or vehicle into the third ventricle. During infusion, rats were subjected to moderate exercise regimens on a treadmill. Exercised mutants receiving K252a exhibited a 21.4% loss in Purkinje cells compared to their controls. Cerebellar TrkB expression was evaluated using non-drug-treated mutants subjected to various treadmill running regimens. Running animals expressed three times more TrkB than sedentary animals. BDNF was quantified via Sandwich ELISA, and cerebellar expression was found to be 26.6% greater in mutant rats on 7-day treadmill exercise regimen compared to 30 days of treadmill exercise. These results suggest that BDNF is involved in mediating exercise-induced neuroprotection.

## 1. Introduction

Cerebellar ataxia is a progressively degenerative and often genetically inherited disorder that is characterized by gait instability, limb rigidity, and difficulties in motor coordination. The cell type most affected in cerebellar ataxia is the GABAergic Purkinje cell [1]. Clinical studies reported that treadmill training for ataxic patients yielded significant improvements in mobility, balance, and gait quality [2, 3]. Other studies using animal models also reported that moderate exercise delayed both the onset and progression of other neurodegenerative diseases in which motor dysfunction is a characteristic manifestation, including Parkinson's [4], ALS [5], and Alzheimer's diseases [6].

The* spastic* Han Wistar (sHW) mutant rat suffers from an excitotoxic neurodegenerative disorder characterized as progressive cerebellar ataxia [7]. Symptoms are visible as early as 25–30 days of age, and anatomical inspection revealed that the mutant undergoes progressive neurodegeneration of cerebellar Purkinje cells [7, 8]. The progression of this autosomal genetic disorder leads to complete loss of motor coordination and shortened lifespan; mutant rats die around 65 days old. Recently, our lab treated young sHW mutants with moderate treadmill exercise performed five days per week over a period of five weeks. Results showed that moderate exercise increased Purkinje cell survival by 62% when compared to sedentary counterparts [9]. As a result of increased cell survival, these rats exhibited significantly improved motor function and increased lifespan. While our initial study corroborated therapeutic research with other animal models and human ataxic patients, the question remains, how does exercise stimulate Purkinje cell neuroprotection in our ataxia model?

Research to date suggests that neurotrophins like brain-derived neurotrophic factor (BDNF) play an important role in mediating this neuroprotection [10]. BDNF's presumptive effect is mediated via receptors that have intracellular domains with intrinsic tyrosine kinase activity. BDNF specifically binds two receptor types: p75^NTR^ to which all neurotrophins bind and TrkB that specifically binds BDNF [11]. Both BDNF and its receptor TrkB are distributed widely throughout the brain [12, 13]. The idea that neurotrophins may mediate the neuroprotective effects of exercise in the brain comes from several studies where increased physical activity elicited increased expression of BDNF [14, 15]. In related studies, BDNF deficient and null mice models helped to prove that gait coordination [16], cerebellar development [17, 18], and function [19] are BDNF-dependent processes.

Our lab has previously shown that moderate treadmill exercise can be effective in attenuating Purkinje cell neurodegeneration observed in the sHW rat, resulting in increased longevity and improved motor function. The goal of this current study was to evaluate the effects of treadmill exercise on the expression and effectiveness of BDNF and its receptor TrkB in this ataxic rat model.

## 2. Materials and Methods

### 2.1. Animals

*Spastic* Han Wistar rats were obtained from the breeding colony in the California State University Northridge (CSUN) vivarium. Animals were housed in standard rat cages and provided Lab Diet 5001 rodent chow and water* ad libitum*. The room temperature was 23°C ± 2°C. Photoperiod was set at 12 h L: 12 h D. Exercised rats were run on a Columbus Instrument Exer 3R motorized treadmill at 15 m/min for 30 min on a 10% slope. The treadmill speed was held constant until the mutant animals could no longer run due to the progression of their disease. Subsequent adjustments in speed and slope were made to allow the aging mutants to run for the entire 30-minute session. Sedentary animals remained in the exercise training facility without being run for the same duration as their exercised littermates. All experiments described in this study have been approved by CSUN's IACUC committee.

### 2.2. Exercise and BDNF Quantification in Various Brain Regions

The expression of BDNF was measured in male mutant and normal littermates (30 days of age) that were randomly divided into one of four activity groups: seven consecutive days of exercise (acute treatment), five exercise days per week for 30 days (chronic treatment), and their seven- or 30-day sedentary counterparts (*n* = 6 per treatment per genotype). Animals were sacrificed rapidly via cervical dislocation within 4 hrs following completion of the final running treatment (either 37 days for the acute treatment or 60 days for chronic treatment). For each animal, the cerebellum, hippocampus, and cortex were extracted and stored at −80°C until quantification, at which time 50 mg of each sample was used to conduct the BDNF sandwich enzyme immunoassay. Each 50 mg sample was sonicated (Fisher Scientific, Model 100 Ultrasonic Dismembrator) in ice cold buffer (100 mM Tris/HCl, 2% BSA, 1 M NaCl, 4 mM EDTA-Na^2+^, 2% Triton X-100, 0.1% sodium azide, 5 *μ*g/mL aprotinin, 0.5 *μ*g/mL antipain, 157 *μ*g/mL benzamidine, 0.1 *μ*g/mL pepstatin A, and 17 *μ*g/mL phenylmethylsulphonyl fluoride). The samples were prepared in 20 times the volume of the 50 mg wet tissue and centrifuged at 14,000 g for 30 min. 50 *μ*L volumes of the supernatants were diluted with 50 *μ*L of the kit's sample diluent and added to the ELISA 96-well plate (Chemikine Sandwich ELISA kit, Millipore) which had been precoated with mouse, anti-Human BDNF monoclonal capture antibody. The plates were incubated on a shaker overnight at approximately 4°C. After 24 hr incubation, well contents were discarded and wells were washed repeatedly with 250 *μ*L volumes of wash buffer. To detect the captured BDNF, 100 *μ*L of biotinylated mouse anti-BDNF monoclonal antibody (1 : 1000) was added to each well and incubated at room temperature on a shaker for three hours. The well contents were again discarded, and the plates were washed with buffer. The plates were then incubated at room temperature for one hour in 100 *μ*L of streptavidin-HRP enzyme conjugate (1 : 1000), and the plates were read immediately using a spectrophotometer microwell plate reader at 450 nm. To determine the concentration of BDNF in the well plates, SoftMax Pro analyzed the absorbance readings as optical density (OD) values by converting them into concentrations (pg/mL), based on the known concentrations of the BDNF standards used in the experiment. Three-way ANOVA was used to test for significant interactions between genotype, sedentary/exercise treatments, and acute/chronic exercise bouts.

### 2.3. K252a Experiment

In order to ascertain whether BDNF activation of TrkB was linked with exercise-induced neuroprotection, rats were given K252a, a known inhibitor of TrkB receptors [19]. Male mutant and normal littermates were randomly assigned to one of four experimental groups: K252a (Tocris Bioscience; normal *n* = 9, mutant *n* = 9) or vehicle 1% dimethylsulfoxide (DMSO) (Sigma Chemical; normal *n* = 10, mutant *n* = 10). Because K252a does not cross the blood-brain barrier, littermate groups underwent intracerebral implantation of an osmotic pump (Alzet model 2004; Durect Corp.) at 28 days of age. This pump chronically delivered the drug or vehicle at a rate of 0.25 *μ*L per hour for the 28-day duration of this experiment. To accomplish pump implantation, the rats were anesthetized intraperitoneally with chloral hydrate (300 mg/kg) and placed on a stereotaxic apparatus. A 3.5 mm cannula attached to the pump was inserted into the third ventricle (−3.3 mm posterior to bregma; 1.0 mm depth). The pumps were placed subcutaneously in the rat's midscapular region and infused K252a at the concentration of 5.2 ng/*μ*L or vehicle (1% DMSO dissolved in 0.9% saline) into the third ventricle. Rats were given a period of 48 hrs of recovery before they began the treadmill exercise regimen. After 28 days of exercise (30 min/day, 5 days/week), animals were anesthetized with 400 mg/kg of chloral hydrate and transcardially perfused with 0.9% saline solution containing 0.2% heparin and 0.1% Na_2_SO_3_ to clear the blood from the vessels in the brain. Next, 4% paraformaldehyde dissolved in 0.1 M phosphate buffered saline (PBS) was used to fix the tissue. Brains were removed and postfixed in 20% sucrose in 0.1 M PBS for 48 hrs prior to sectioning. The cerebellum was sliced into 30 *μ*m sagittal sections using a cryostat. Only rats where correct cannula placement was verified were used for subsequent cresyl violet histological examination. Slides containing the cerebellar sections were hydrated in a series of ethanol solutions (100%, 95%, and 70%) and distilled water for 2 min each. The slides were then submerged in cresyl violet followed by differentiation with acetic formalin (0.2% glacial acetic acid and 4% formaldehyde in deionized H_2_O), rinsed in distilled water, and dehydrated in a series of ethanol solutions (95% and 100% EtOH). Finally, sections were cleared in xylenes and coverslipped using Permount for preservation of tissue.

### 2.4. Purkinje Cell Counts

Nissl stained sections were used to evaluate the number of Purkinje cells present in the four treatment groups: normal and mutant K252a and normal and mutant DMSO (vehicle) treated runners. Two observers, who were blind to treatment, performed the cell counts using an Olympus microscope at 400x magnification. Three randomly selected Purkinje cell lobule areas (per 300 *μ*m transect) were counted for a total of ten sections per animal and were analyzed at the same stereologic level for all animals. Since there are approximately 20 sections per animal (10 sections per animal on two different slides), we are convinced that just by random chance each counter would have a less than 1% chance of counting the exact same section. The dependent variable “cell count” was computed by averaging the total number of Purkinje cells for each of the sections counted so that an average Purkinje cell count number per 300 *μ*m transect was determined for each animal. Cell counts were analyzed by two-factor ANOVA to test for significant differences between the normal and mutant genotypes treated with K252a or vehicle.

### 2.5. Motor Activity Test

K252a treated animals were tested weekly in an open field arena just prior to treadmill running. The activity box arena (100 × 100 × 35.5 cm) was constructed out of acrylonitrile butadiene styrene, and a digital camera, hung directly above the activity box arena, recorded the movement of the animals during each trial. The test consisted of three nonconsecutive trials, each lasting two minutes with 2-3 min of rest between trials. Rodent tracking software* Rodent Tracker 3000* was used to determine the distance traveled (cm) by each rat. All activity video files were uploaded, and the open field arena and subject (moving rat) were calibrated for placement. The program then examined each frame of the video and determined the total distance traveled (cm) within the two-minute time period. The distance traveled in each trial was averaged and used as the mean activity score for each animal at ages 30, 37, 44, and 51 days. The mean activity scores were first log-transformed to achieve normal distribution and then analyzed using repeated measures ANOVA to test for significant differences in the rate of decline in normal and mutant genotypes treated with K252a and vehicle.

### 2.6. Treadmill Exercise and TrkB Immunostaining

In order to examine whether TrkB receptor expression is linked with the neuroprotective effects of exercise in the cerebellum, male mutant and normal littermates (*n* = 6 per treatment group) were exposed to the same four treatment regimens listed above (Section 2.2). Acutely exercised and sedentary groups were transcardially perfused with 4% paraformaldehyde at 36 days old while the chronically treated groups were perfused at 60 days old, as described above (Section 2.3). Cerebellar sections (30 *μ*m) were immunostained with anti-TrkB (Millipore; dilution 1 : 3000 in serum block buffer) using standard immunohistochemical procedures. Briefly, tissue sections were first washed in three changes of 0.1 M PBS for 5 min each and subsequently treated with 0.3% H_2_O_2_ to block endogenous tissue peroxidase. After 30 min in hydrogen peroxide, sections were rinsed in three changes of PBS and then incubated in serum block (5% NGS and 0.2% Triton-X in 0.1 M PBS) for one hour to inhibit nonspecific binding. The cerebellar sections were transferred directly from serum block into wells containing anti-TrkB primary antibody and incubated on a shaker at 4°C for 24 hrs. Following incubation in primary antibody, sections were rinsed with three changes of PBS and incubated at room temperature in wells containing the biotinylated rabbit IgG secondary antibody for two hours (ELITE ABC kit, Vector Laboratories; diluted in 1.5% NGS, 0.2% Triton-X/0.1 M PBS). Sections were then rinsed in three changes of PBS, transferred to wells containing the avidin-biotin complex (ELITE ABC kit, Vector Laboratories), and incubated at room temperature for one hour. At the end of the incubation, sections were once again rinsed in three changes of PBS and treated with the chromogen 3,3′-diaminobenzidine for 3–5 min. Sections were washed in three changes of 0.1 M PBS, placed onto glass slides, and allowed to air-dry for 24 hrs. After drying, sections were dehydrated in a series of ethanol solutions (95% and 100% EtOH), cleared in xylene, and coverslipped using Permount. All cerebellar sections were analyzed at the same stereologic level for all animals and blindly scored from 0 to 3 based on the intensity of antibody staining in the Purkinje cell (PC) layer in comparison to the surrounding granule cell (GC) and molecular cell (MC) layers. If the PC layer appeared lighter than the surrounding GC and MC layers, a “0” was given denoting no TrkB staining. The presence or absence of TrkB staining was confirmed by comparison to sections that were intentionally left untreated with the TrkB primary antibody for use as a negative control. A score of “1” was assigned to sections where the PC layer blended with the surrounding cerebellar layers. Scores of “2” and “3” were assigned to sections where the PC layer was darker or much darker than the surrounding layers. Two sections were averaged for each of six animals in the eight treatment groups. A three-way ANOVA was used to test for significant interactions between genotype, sedentary/exercised treatments, and acute/chronic exercise regimens.

## 3. Results

### 3.1. Exercise and BDNF Quantification in Various Brain Regions

#### 3.1.1. Cerebellum (Figure 1(a))

Three-way ANOVA showed there were no significant three-way or two-way interactions on BDNF expression (*P* > 0.25). However, there was a significant difference in BDNF concentration (pg/mL) between acutely and chronically run animals (*F* = 7.58; *P* < 0.05) regardless of the genotype. Yet, there were no significant effects of genotype (*F* = 0.121; *P* > 0.05) or running treatment (*F* = 1.54; *P* > 0.05) alone on BDNF concentration in the cerebellum.

#### 3.1.2. Hippocampus (Figure 1(b))

The hippocampus had the highest concentration of BDNF in our study. The three-way and two-way interactions were not significant (*P* > 0.25). However, unlike within the cerebellum, a significant difference in BDNF concentration between running and sedentary animals was found (*F* = 4.47; *P* < 0.05). Like the cerebellum, the effect of genotype on BDNF concentration was insignificant (*F* = 3.30; *P* > 0.05) in the hippocampus. There was no significant difference found between chronic and acute treatments (*F* = 0.067; *P* > 0.05).

#### 3.1.3. Cortex (Figure 1(c))

Acute running increased BDNF concentration in the cortex, which contained the lowest levels of BDNF in our study. Three-way ANOVA showed there were no significant interactions of genotype × sedentary/exercised treatments × acute/chronic treatments on cortical BDNF concentration. Statistically significant differences in BDNF concentration between running and sedentary animals (*F* = 6.91; *P* < 0.05) and between the 7-day and 30-day treatment durations (*F* = 4.54; *P* < 0.05) were found. There was no effect of genotype on BDNF expression (*F* = 1.90; *P* > 0.05) in the cortex.

### 3.2. K252a Experiment

#### 3.2.1. Purkinje Cell Counts

To determine whether exercise-induced neuroprotection involved BDNF, the TrkB inhibitor K252a was administered. A two-factor ANOVA showed a significant interaction between genotype and drug treatment (*F* = 4.87; *P* < 0.05). Further quantitative analysis of mean Purkinje cell (PC) counts (Figure 2) revealed that mutant rats had significantly greater PC loss than normal rats regardless of treatment (*F* = 6.63; *P* < 0.01). Within the mutant groups, exercised K252a treated mutants averaged a statistically significant 21.4% fewer PCs than exercised DMSO-administered mutants (*t* = 3.18; *P* < 0.01). There were no differences between drug- and vehicle-treated normal siblings (*t* = 0.28; *P* > 0.05).

Examining representative cerebellar sections (Figure 3), there were no apparent differences in PC number between DMSO and K252a treated normals. However, there were obvious differences between DMSO and K252a treated mutants. Note the paucity of PCs in the mutant rats treated with the TrkB antagonist, K252a. Although DMSO mutants had more PCs, the PCs still appeared somewhat necrotic in contrast to the round shape seen in the normal animals. Cell density and morphology within the granular and molecular cell layers did not appear to change across drug treatment or genotype. Also, no differences in total cerebellar volumes were observed among the four treatment groups.

#### 3.2.2. Activity Testing

Motor activity was measured weekly during the 28-day running and drug-infusion period (Figure 4). The mean distances traveled were log-transformed, and repeated measures ANOVA was used to evaluate the effects of age considering genotype and treatment. The statistical analysis showed no significant interaction between treatment and specific genotype. DMSO and K252a treated normal rats exhibited no significant decline in activity over the four-week period (*F* = 0.73; *P* > 0.05). However, mutant rats regardless of treatment had lower activity, averaging a total distance traveled of 572.25 cm compared to the average distance traveled by normal rats, 885.89 cm (Figures 4(a) and 4(b)). While mutants treated with K252a exhibited the greatest progressive decline in activity with age, this decline was only marginal (*F* = 2.40; *P* = 0.08).

#### 3.2.3. Exercise and TrkB Immunostaining

To investigate whether TrkB receptor expression could be influenced by BNDF's rise during exercise, 30-day-old mutant and normal animals were subjected to either 7 days (acute) or 30 days (chronic) of running, and control animals were kept sedentary for their respective lengths of time. Cerebellar sections were stained with anti-TrkB primary antibody, and TrkB expression was quantified and analyzed by three-way ANOVA. Figures 5, 6, and 7 show that acute exercise resulted in a greater expression of TrkB than what was found in the sedentary controls and in animals that were in the chronically exercised group. Quantitatively, there was a significant three-way interaction (genotype × sedentary/exercised treatments × acute/chronic exercise bouts) concerning TrkB expression (*F* = 4.24; *P* < 0.05). A synergistic effect was also significant for sedentary-versus-exercised × acute-versus-chronic exercise (*F* = 4.44; *P* < 0.05). In addition, normal animals consistently expressed more TrkB than mutants in the cerebellum (*F* = 22.3; *P* < 0.01), and animals that were exercised expressed significantly more TrkB than their sedentary counterparts (*F* = 16.0; *P* < 0.01). Because there was a significant three-way interaction, separate two-way ANOVAs were performed for both the 7-day and 30-day treated animals to further evaluate the genotype × sedentary/exercised interactions. In the acutely treated animals, a significant effect of both genotype (normal v mutant) (*F* = 14.1; *P* < 0.01) and exercise treatment (*F* = 20.5; *P* < 0.01) on TrkB expression was detected. In the chronically treated animals, a significant genotype × sedentary/exercised treatment interaction was revealed (*F* = 6.98; *P* < 0.05). Additionally, the effect of genotype on TrkB expression in this group was greater than the effect of exercise treatment (*F* = 9.26; *P* < 0.01).

## 4. Discussion

**Figure 1:**
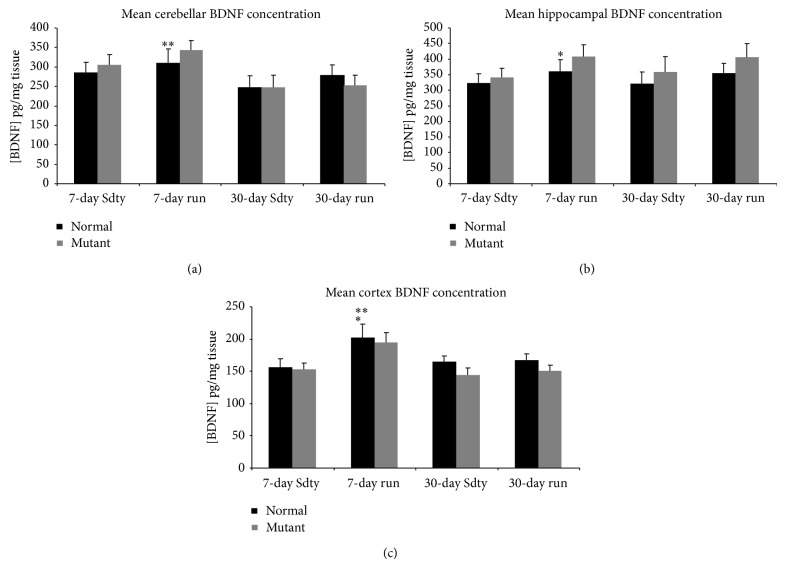
BDNF concentration in the cerebellum (a), hippocampus (b), and cortex (c) of rats exposed to sedentary (Sdty), acute (7-day run), or chronic (30-day run) running regimens (*n* = 6 for each treatment in each brain region). The three-way ANOVA revealed no significant three-way or two-way interactions. However, there was a significant effect of treatment duration on BDNF concentration in both cerebellar and cortical regions of acute runners compared with chronically run rats (^**^ *P* < 0.01 and *P* < 0.05, resp.). There was a significant effect of running on hippocampal and cortical BDNF levels compared to their sedentary sibling counterparts (^*^ *P* < 0.05). Values are mean ± SEM.

**Figure 2:**
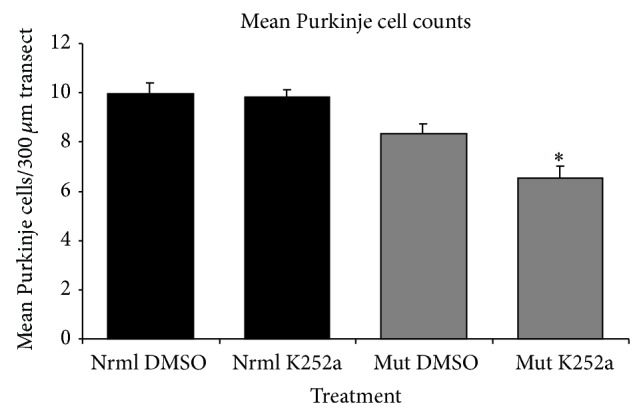
Mean Purkinje cell counts in the cerebellums of exercised mutant (Mut) and normal (Nrml)* spastic* Han Wistar rats (*n* = 9 pairs) after 28-day treatment with K252a (TrkB antagonist) or DMSO (vehicle). Two-factor ANOVA showed a significant synergistic effect of genotype and treatment. Asterisk indicates that mutant animals treated with K252a had significantly fewer Purkinje cells surviving than DMSO-treated mutants. There were statistically similar numbers of Purkinje cells in normal rats regardless of the treatment. Values are mean ± SEM.

**Figure 3:**
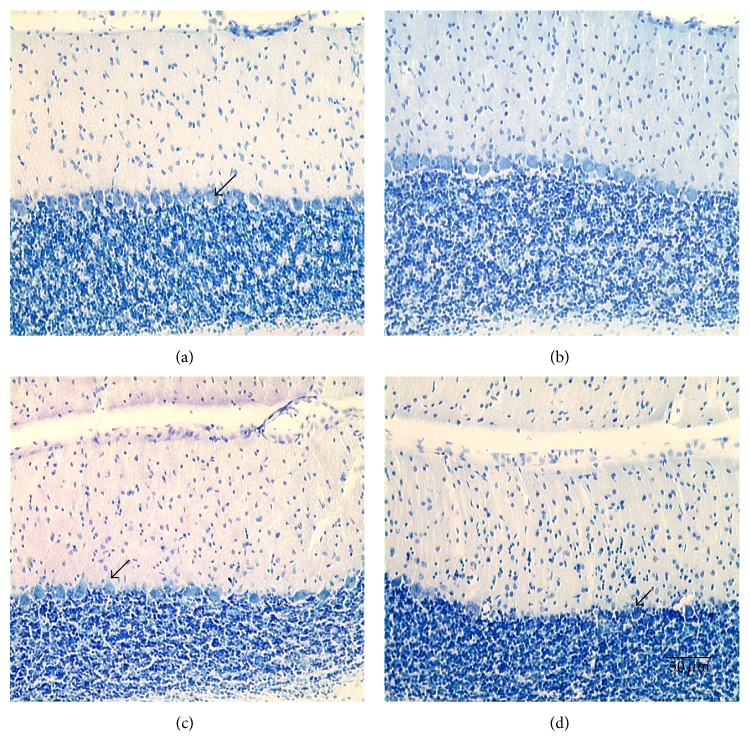
Photomicrographs of cerebellums from normal animals treated with (a) DMSO (vehicle) or (b) K252a and mutant animals treated with (c) DMSO or (d) K252a. All animals were subjected to a 28-day running regimen. Note the slight loss of Purkinje cells in mutant (c) (DMSO-treated) compared to the significant loss in the K252a treated mutants (d). Arrows indicate surviving Purkinje cells. Scale bar in the lower right panel refers to all photos (50 *μ*m at 200x magnification).

**Figure 4:**
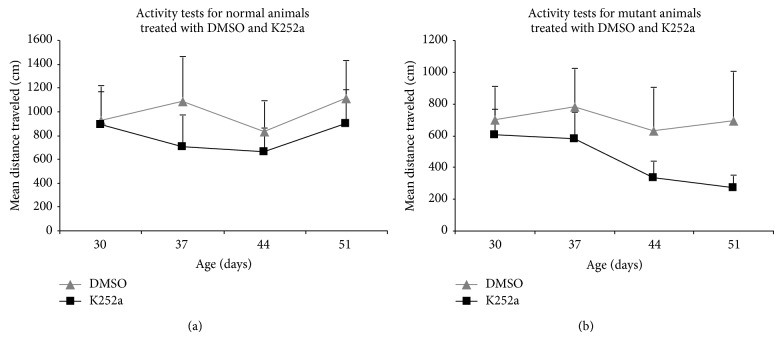
Mean distance traveled for (a) normal and (b) mutant rats treated with either K252a or DMSO (*n* = 9) and subjected to a chronic exercise regimen (28 days). Activity testing was performed once a week over the 28-day treatment period. Repeated measures ANOVA showed that the decline in activity was not dependent on treatment. The genotype had a slight but nonsignificant effect on the activity as the animals aged. Values are mean ± SEM.

The main question addressed in this study was whether BDNF was involved in exercised-induced neuroprotection of Purkinje cells in the* spastic* Han Wistar rat (sHW), a phenomenon that we have recently observed with aerobic treadmill running [9]. We found several lines of evidence linking BDNF and its receptor TrkB with exercise-induced neuroprotection in the cerebellum of the sHW rat. The most obvious was that BDNF expression was elevated within one week of moderate exercise (Figure 1) and corresponded to the most susceptible period in Purkinje cell survival in the sHW mutants. Previous studies performed in our lab showed that untreated exercising mutants began showing significant declines in motor activity around 40 days of age [9]. These results were corroborated by the administration of a known TrkB antagonist, K252a, that blocked Purkinje cell survival in exercising rats. Exercising animals treated with K252a had significantly fewer surviving Purkinje cells (Figures 2 and 3). The progressive decline in motor activity of K252a treated mutants beginning around 37 days of age (Figure 4) corroborates the potential role of BDNF in exercise-induced neuroprotection of Purkinje cells.

**Figure 5:**
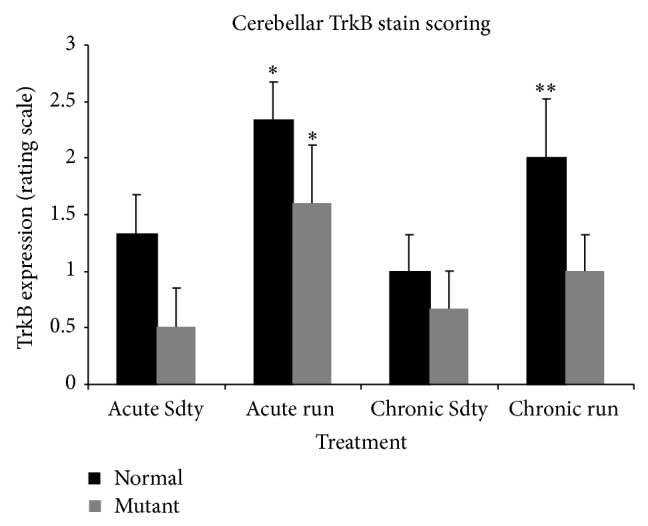
TrkB expression in the cerebellums of acutely exercised (7 days), chronically exercised (30-day run), and their sedentary (Sdty) mutant and normal controls (*n* = 6 per training regimen and genotype). Three-way ANOVA showed a significant interaction of genotype × exercise × treatments (*P* < 0.05). Significant increases in TrkB expression were observed in acute runners compared to their sedentary counterparts (^*^ *P* < 0.01). In the chronically treated group, the genotype (^**^ *P* < 0.01) had a greater effect on TrkB expression than running treatment (*P* > 0.05). A synergistic effect of running and genotype (normal) on TrkB expression was found in the chronically treated group (^*^ *P* < 0.02) and *y*-axis values were based on a scoring intensity ranking of TrkB immunostaining. Values are mean ± SEM.

**Figure 6:**
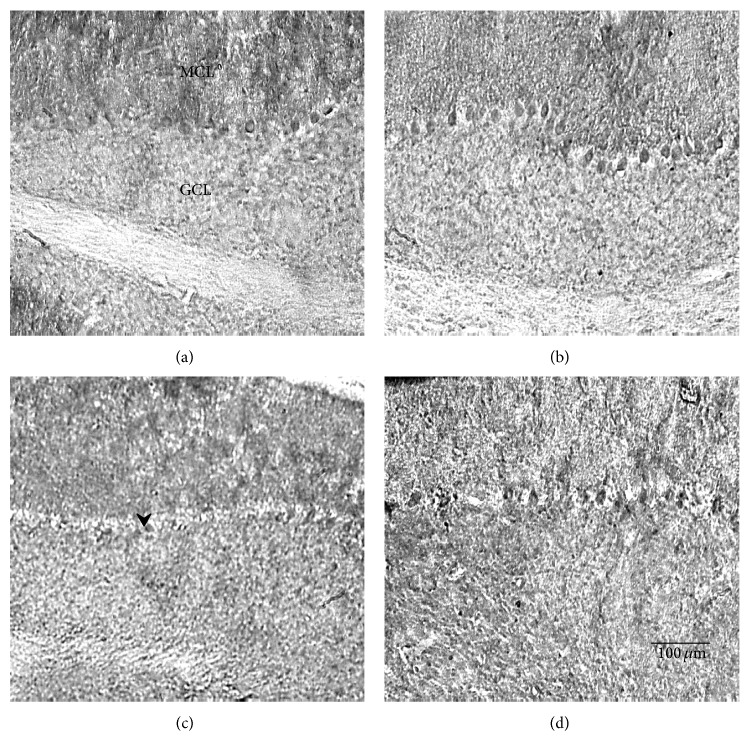
Photomicrographs of the TrkB stained cerebellums from mutant animals exercised for 7 days (b) or 30 days (d) and their sedentary controls (a) and (c), respectively. Notice that acutely treated runners in (b) have significantly darker stained Purkinje cells than sedentary controls in (a). MCL: molecular cell layer; GCL: granule cell layer. Scale bar in the lower right panel refers to all photos.

**Figure 7:**
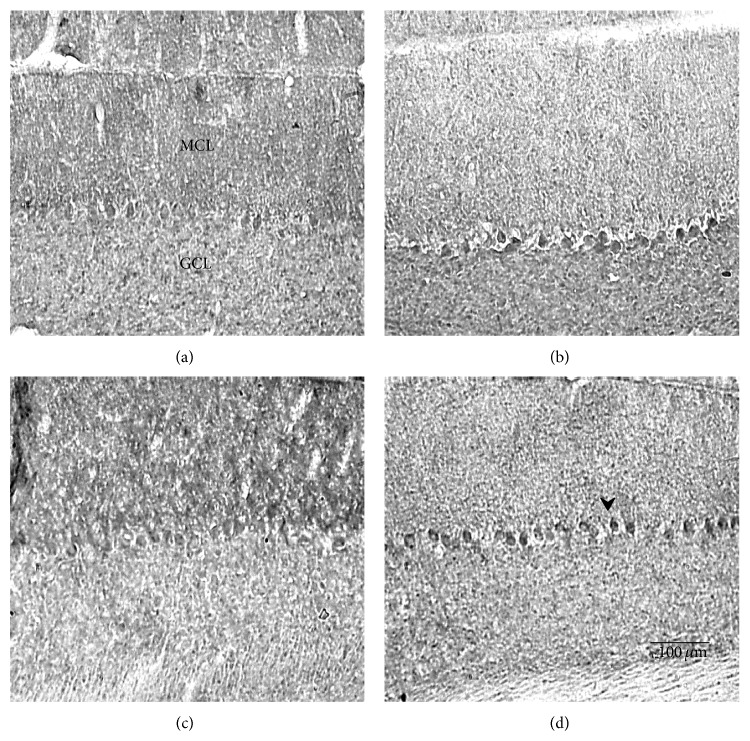
Photomicrographs of the TrkB stained cerebellums from normal animals exercised for 7 days (b) or 30 days (d) and their sedentary controls (a) and (c), respectively. Notice that runners regardless of the 7-day (b) or 30-day (d) exercise duration have darker stained Purkinje cells than the sedentary controls (a) and (c), respectively. MCL: molecular cell layer; GCL: granule cell layer. Scale bar in the lower right panel refers to all photos.

One explanation linking BDNF, TrkB, and Purkinje cell neuroprotection is a possible alteration in cerebellar TrkB receptor expression with exercise. Immunohistochemical analyses revealed that cerebellar TrkB expression was significantly higher in running animals than in sedentary animals, regardless of the treatment duration (Figure 5). These data suggest a synergistic interaction of the genotype, exercise treatment, and exercise duration such that normal animals on an acute running regimen expressed the highest concentrations of TrkB and chronically sedentary mutants expressed the least concentrations. These data contradict the results reported by Klintsova et al. [12] where no significant differences in TrkB protein levels were found to exist between exercised animals and controls after two weeks of moderate exercise. It should be noted that Klintsova et al. [12] quantified the full-length isoform of the TrkB receptor, whereas no distinction between isoforms was made in our experiment. However, in this study, as well as the Klintsova et al. study, TrkB expression was elevated within seven days of running.

It was expected that BDNF levels would increase concomitant with the increased TrkB expression. Cerebellar BDNF levels instead appeared to remain constant across the different treatment groups (Figure 1). Only acutely exercised mutants expressed significantly more BDNF than chronically exercised mutants, but not significantly more than their sedentary counterparts. Normal acutely run animals also did not display a significant increase in cerebellar BDNF levels when compared to their sedentary littermates. The fact that acutely run animals expressed more BDNF and TrkB than chronically run animals suggests that BDNF and TrkB expression change as the animals age and adapt to a regular, chronic exercise paradigm. This phenomenon has been shown by other researchers.

BDNF levels are known to vary with both exercise duration and vigor [12, 20]. Klintsova et al. [12] used 35-day-old female Long-Evans rats to study changes in BDNF and TrkB expression in response to exercise over two weeks. Like our study, they reported that moderate physical activity increased BDNF and TrkB expression in the cerebellum within seven days. However, they reported no significant differences in TrkB or BDNF protein levels after 14 days of exercise and suggested that changes may have occurred within the first week of activity after which time the repeated experience of exercise on a track may not be enough to cause further increases [12]. Ploughman et al. [20] compared hippocampal BDNF levels between a forced 60-minute motorized run (higher intensity) and 12 hours of voluntary wheel running (lower intensity) over a two-week duration in animals that had been exposed to ischemic injury. These researchers reported that forced exercise treatment resulted in increased BDNF levels with short-lived expression, while the voluntary, low intensity wheel running resulted in longer lasting expression of BDNF.

In the cortex and hippocampus, acutely exercised mutants expressed more BDNF than their sedentary counterparts (Figure 1). Similar to the cerebellum, BDNF protein expression in the cortex of acutely exercised mutants was also significantly greater than chronically exercised mutants. The hippocampus had the highest concentration of BDNF, while the cortex had the least concentration. This is not surprising given that a major function of BDNF in the adult rat brain is neuronal plasticity, including long-term potentiation in the hippocampus [21]. Another related study quantified BDNF expression with treadmill exercise in Wistar rats and reported similar cortex < cerebellum < hippocampus BDNF expression results [22]. Cechetti et al. [22] used adult rats (aged 2-3 months) on a treadmill regimen, and, just as with normal animals in the current study, they found no significant increases in BDNF within the hippocampus, cortex, or cerebellum following chronic exercise.

Hopkins et al. [23] reported differences in BDNF expression in adolescent and adult, exercised and nonexercised Long-Evans rats. These researchers reported that exercise increased BDNF levels in the cortex and hippocampus in adult rats, but expression began declining to baseline two weeks after exercise ceased. However, akin to the chronically treated rats reported in this study [23], the adolescent rats were subjected to four weeks of running regimens beginning at 32 days old, showing no significant differences in BDNF levels between exercising and nonexercising groups immediately following the treatment period. It should be noted that Hopkins' rats [23] were subjected to voluntary wheel running regimens as opposed to the forced treadmill regimen used in our study.

Das et al. [24] studied the developmental patterns and differential expression of BDNF in nonexercised rats and reported that levels increased as the animals aged, peaking in the hippocampus, neocortex, and cerebellum between 7 and 21 days. After this point, BDNF levels either plateaued or decreased slightly as the animals aged further. Since the animals in this study began running treatment at 30 days of age, it is likely that a higher intensity exercise paradigm, or one where intensity was gradually increased, was required to cause a lasting significant increase in BDNF expression in chronically exercised animals compared to sedentary controls. However, in the case of the cerebellum and cortex, acutely exercised mutants (30–36 days of age) still expressed significantly higher levels of BDNF than chronically exercised mutants. Because previous work done in our lab showed that mutants had an accelerated physical decline beginning around 40 days old, the increase in BDNF in acutely exercised mutants at 30 days of age may signify a critical time point when destabilizing neurologic changes begin to modify the mutant cerebellum.

Adlard et al. [6] also studied the time course of exercise-induced BDNF expression in the hippocampus. They reported that increased wheel running distance was correlated with increased BDNF expression. The variation in BDNF expression across the aforementioned studies including the present study may be the result of changes in the expression ratio of multiple BDNF mRNA transcripts. Adlard et al. [6] addressed this possible change in transcript expression across time points and concluded that the exon encoding the mature BDNF protein was significantly upregulated in exercising animals when compared with nonexercising counterparts. This finding was also observed comparing exercised wild-type mice with those that were heterozygous for the BDNF gene [25]. Therefore, various exercise paradigms may result in varying BDNF expression patterns, depending on the type of exercise paradigm, duration of treatment, and age. Future work exploring the relative amount of BDNF mRNA produced in exercising animals may provide a better explanation for how BDNF functions in cerebellar neuronal protection.

There are a number of ways in which BDNF could improve neuroprotection for susceptible neurons like Purkinje cells. BDNF triggers the activation of both the phosphatidylinositol-3-kinase (PI3K) and Ras-MAPK pathways that result in the transcription of genes that augment cell survival via antiapoptotic means [26]. Activation of the MAPK pathway triggers phosphorylation of CREB resulting in the expression of antiapoptotic proteins and suppression of the proapoptotic proteins, Bcl-2 and BAD, respectively [27, 28]. CREB is also thought to be a regulator of BDNF gene expression [29]. Activation of the PI3K pathway results in the expression of certain protein kinases, PKB [26] and PKC [30]. PKB was shown to suppress the actions of caspase-3, an essential mediator of apoptosis [31], while PKC is thought to phosphorylate gene transcription factors as well as activate the MAPK pathway [30, 32]. However, the neuroprotective actions of BDNF probably extend beyond apoptosis suppression.

Purkinje cells are quite susceptible to excitotoxicity perhaps due to having two excitatory (glutamatergic) inputs, parallel fibers and climbing fibers [33, 34]. Purkinje cell death and the concomitant ataxic symptoms in the* spastic* Han Wistar mutant may be due to subtle biochemical changes within these neurons rather than solely from glutamate excitotoxicity. For this reason, it would be useful to identify changes in various intracellular enzymes and proteins with regard to BDNF production in exercising mutants. It may also prove useful to investigate whether acute bursts of exercise over the chronic treatment duration would induce any spatial or temporal changes in BDNF expression. Perhaps this proposed experiment would offer a better explanation of how early physical activity stimulates the greatest level of BDNF synthesis leading to Purkinje cell neuroprotection.

## 5. Conclusions

This paper addresses whether BDNF is involved in exercise-induced neuroprotection of Purkinje cells previously correlated with aerobic treadmill running [9]. Our study found several novel lines of evidence linking BDNF-TrkB pathway with exercise-induced neuroprotection in the cerebellum of the* spastic* Han Wistar rat, a model of ataxia. For example, these results demonstrated that it was possible to block Purkinje cell survival of exercising rats by the administration of a known TrkB antagonist, K252a. We hope that our studies of this ataxic rat model will contribute to an increased understanding of incorporating behavioral modifications, like moderate exercise, into treatment paradigms for diseases like ataxia.
